# COVID-19 Preventive Measure: A Study on “MASK-A Boon or a Bane”

**DOI:** 10.1155/2022/2253656

**Published:** 2022-09-16

**Authors:** Revati Amin, Ranita Ghosh Dastidar, Vaishali K

**Affiliations:** ^1^Department of Physiotherapy, Manipal College of Health Professions, Manipal Academy of Higher Education, Manipal, KA 576104, India; ^2^Department of Biochemistry, Kasturba Medical College Manipal, Manipal Academy of Higher Education (Institute of Eminence), Manipal, KA 576104, India

## Abstract

**Background:**

Mask wearing can restrict the spread of respiratory viral transmission during the third wave of the COVID-19 pandemic. Globally, governments have emphasized its use in workplaces and public areas to prevent the transmission routes of corona virus. In spite of the current awareness in the general population, the stringency of wearing the mask lies as an individual's choices. *Subject and Methods*. This research work discusses available studies on the types and comparison of masks in the market for use. It includes a short survey conducted with 1,173 anonymized healthy participants primarily devoid of comorbidities. The survey includes the effects of mask wearing, while outdoor with minimal activities like walking and with mild activities like jogging and stretching. Our research further discusses various health effects of wearing a mask, including cardiac output, hypoxemia, hypoxia, and dyspnoea, and how such situations that pose a threat can be consciously avoided.

**Results:**

It was found that the majority of people use cloth/fabric reusable mask. There remains scope for better-designed masks and improving health in the mass population by inculcating healthy breathing habits and other relevant exercises that can help people cope up better in this fight against the deadly virus at a larger scale.

**Conclusion:**

For most of the survey questions, there was significant correlation between gender and the related responses as there was no significantly observable difference in the nonparametric, unpaired analyses of responses. The main objective of this research work is to initiate more discussions and enhance awareness in natural ways of staying healthy during the pandemic emphasizing mask use. Further progress in this aspect remains a whole new area for future exploration.

## 1. Introduction

Coronavirus 2019 (COVID-19) is the second influenza A H1N1 pandemic in the twenty-first century, following the 2009 pandemic caused by severe acute coronavirus 2 respiratory syndrome (SARS-CoV-2). Coronavirus 2019 (COVID-19) is closely linked to coronaviruses related to SARS bat 1 [1–3]. World Health Organization (WHO) proclaimed COVID-19 was the first pandemic caused by coronavirus on March 11, 2020 [4]. By June 14, 2020, over 1 million people in 216 nations would have been infected, with over 7,690,708 documented cases and 427,630 fatalities globally. On March 24, 2020, the Indian government declared a 21-day state-wide lockdown as a precautionary measure [5, 6]. The wearing of a facemask has been made a requirement in order to get basic essentials [7]. The government issued Unlock 1.0 on June 1, 2020, for 30 days. The 7^th^ human corona virus known as SARS-CoV-2 is a highly infectious virus that spreads expeditiously between individuals through four different routes: faeco-oral openings, physical contact, aerosol droplets, and airborne passages [8–12]. The WHO recommends five steps [13] to be strictly followed globally as preventive measures against COVID-19. The lockdown is in process with the second wave. These include the following:

- Maintaining personal hygiene, washing hands with soap and water, using alcohol-based hand sanitizers, and wearing face mask,
- Cleaning surfaces such as door knobs and handles, furniture's, and clothes wallets,
- Maintenance of physical and social distancing in public places,
- Tracking the spread of this virus,
- Having provision for sufficient testing.

Four types of vaccines are being used such as mRNA vaccines, which are genetic compounds of viral proteins; standard vaccines using attenuated strains of the virus; injecting the virus into the backbone of the other viruses; and laboratory techniques using adjuvants [14].

Various medications are under trial and use for prevention and cure against COVID-19 that include antimalarial drug hydroxychloroquine and plasma transfusion from asymptomatic individuals [15, 16].

Wearing a face mask in both hospitals and communities has become ubiquitous during the COVID-19 pandemic. COVID-19 transmission routes include direct (droplet, coughing, and sneezing) and contact (oronasal and mucous membranes of the eye) transmission routes [17]. A significant research subject for the use of mask in public is the control of droplets source.

This effectively blocks the droplets from an infectious person, particularly during speech, when droplets are expelled at a lower pressure and are not small enough to squeeze through the weave of a cotton mask [18–20].

While exercise is critical for the maintenance of sound health, the addition of a mask poses a range of safety issues [21]. First of all, the face cover will limit the flow of air. Mask tolerance, particularly for people with lower lung function, is more important than people realize [22]. Physical exercise will make a mask unbearable that is otherwise tolerable to ventilation [23].

### 1.1. Types of Masks Available

Medical masks are a kind of personal protection equipment that is used to prevent respiratory infections from spreading. These masks cover the wearer's breath and nose and can help prevent respiratory viruses and bacteria from being transmitted, if worn properly. Some commonly available masks are enlisted in Table 1.

A minute volume that represents the volume of air expired in one minute is the standard breathing measure for measuring breathing efficiency [24]. During rest, minute respiration volumes normally range from 5 to 8 L/min. Minute ventilation increases semilinearly from rest to maximum levels with increased exercise rates. In severe cases, ventilation levels of up to 200 L/min1 were recorded [17, 24]. During both progressive and high-intensity constant load work, ventilation appears to decline significantly in PE at work intensities around 80%, PO_2_ max wearing APR, compared with unmasked conditions [25, 26], and VE max values appearing to be 24% lower than unmasked values during incremental exhaustive load work with a full-face piece APR. Others found reductions in PE values on the order of about 15% to 43% under high-intensity constant load work conditions with similar respirator [17].

### 1.2. Extended Use and Limited Reuse

The N95 respiratory masks are strictly for single use only and should be the standard protocol as far as possible. Secondarily, FFP2 masks can be used when N95 masks are unavailable. N99, N100, and FFP3 are not very convenient to wear for extended periods, making it difficult to breathe and therefore are not recommended for a virus filtering [27].

The close fit and filtration properties are essential for extended use and reuse. Sterilizing must eliminate the virus's risk, be safe, and protect respiratory integrity. No sharing is permissible; each person must always wear his or her personal mask. One must discard the N95 mask when it becomes soiled or damaged or when breathing becomes difficult owing to a blocked filter. An individual must not reuse the soiled mask [28, 29].

In the light of the pandemic, despite vaccinations, the spread of COVID-19 is not under control in a few developing nations, such as India. When people are outside their homes, wearing a face mask has become a necessity and a part of their daily routine. The study's objectives included investigating the general healthy population's comprehension of the need for and repercussions of wearing a facemask, as well as their attitude towards physical activity and adaptation in their daily routine, during COVID-19.

## 2. Methodology

### 2.1. Selection Criteria

The inclusion criteria were all types of articles related only to humans and articles published in PubMed. The exclusion criteria were those papers not published in English language and grey literature. The authors identified additional references from the articles retrieved in the initial search round by a manual search among the cited references.

### 2.2. Search terms

Terms to describe masks are as follows: mask, face mask, surgical mask, cloth mask, respirators, respirator mask, respiratory protective devices, air purifier mask, protective face mask, N95, air-purifying respirators, and fabric mask.

Terms to describe exercise training are as follows: exercise, exercise training, aerobic exercises, aerobic training, aerobic exercise training, resistance training, resistance exercise, resistance exercise training, flexibility exercise, fitness, health performance OR physical limitation, outdoor exercise, hypoxia, and fitness.

Terms to describe COVID are as follows: COVID, COVID-19, pandemic, COVID pandemic, and COVID-19 pandemic.

### 2.3. Search Strategy

**Figure 1:**
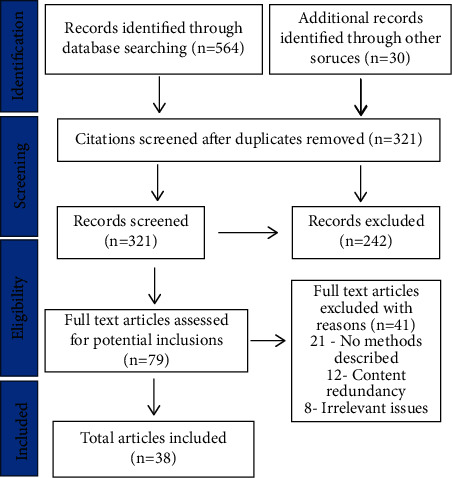
Representation of search strategy used to screen and finalize articles that were used to review various types of masks, their usage and best suited mask types that are already published in records.

**Figure 2:**
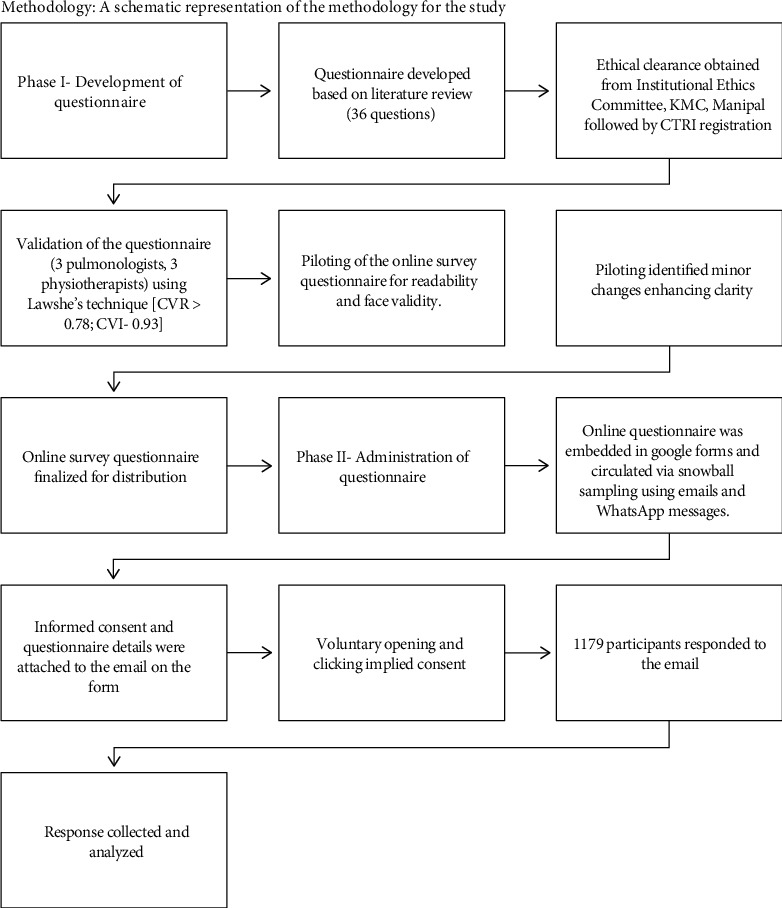
Methodology: A schematic representation of the methodology for the study.

An initial electronic search of PubMed was undertaken followed by the analysis of the text words contained in the title and abstract of retrieved papers and of the index terms used to describe the articles. All identified keywords and index terms were undertaken across all included databases. The reference lists of all included articles and reports were searched to identify additional relevant information sources. In addition, hand searching in journals was performed. Articles were assessed for inclusion based on the inclusion criteria, examining them by title and abstract. If an article met the inclusion criteria, or if further examination of the article was required before exclusion, the full text was retrieved (Figure 1). Methodology is depicted in Figure 2.

### 2.4. Study on Mask Use

A survey questionnaire was developed and circulated among the mass population. The methodology was divided into 2 phases: development of the questionnaire and administration of the questionnaire.

Phase 1: the questionnaire consisted of 36 questions and was developed based on literature review. Permission was obtained from the Institutional Ethics Committee Kasturba Medical College and Hospital, MAHE. CTRI approval undertaken for survey: CTRI/2020/08/027046. The questionnaire was validated using Lawshe's technique for content validation by a panel of 6 experts (3 pulmonologists and 3 physiotherapists). Questions with content validity ratio (CVR) of equal to or more than 0.78 were retained, and content validity index (CVI) of 0.93 was obtained for the designed questionnaire “Questionnaire on use of mask: knowledge, attitude, and practice among individuals during COVID-19.” 5 independent healthcare professionals piloted the online survey for readability and face validity. Piloting identified minor changes requiring modifications for enhancing clarity on a couple of questions, and the online survey was finalized for distribution.

Phase 2: the Google form questionnaire was circulated among the mass population via snowball sampling. It was circulated via e-mails and WhatsApp messages. Questionnaire details and link to informed consent were attached to the e-mail. Voluntary opening and clicking the link implied consent.

### 2.5. Methodology: A Schematic Representation of the Methodology for the Study

A total of 1173 participants responded to the survey questionnaire. Participants above 16 years of age belonging to any gender, who are able to read and understand English and able to ambulate, nonfebrile, and having no musculoskeletal and neurological conditions that would limit their mobility, were included in the study. The response was collected, and analysis was performed using GraphPad Prism 9 software.

## 3. Results

**Figure 3:**
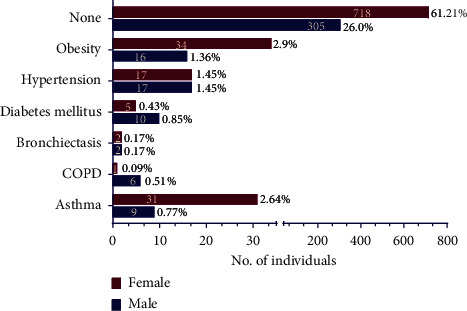
Comorbidities in the number of participating individuals. The comorbidities included in the questionnaire are obesity, hypertension, diabetes mellitus, bronchiectasis, chronic obstructive pulmonary disease, and asthma. Numbers inside the bar graphs represent total number of individuals, while numbers outside the bar graphs represent percentage of total participants. The graph depicts that the survey included participants majorly healthy.

A total of 1173 participants (808 women and 365 men) voluntarily agreed and filled the Google form questionnaire. Hence, this survey included a majority of 69% of female participants. The questionnaire was circulated among the general healthy population that is evident from our data showing a meagre 150 people suffering from any comorbidities such as obesity, hypertension, diabetes mellitus, bronchiectasis, chronic obstructive pulmonary disease, and asthma (Figure 3). This is merely 12.8% of the total participants.

To summarize the responses regarding the current knowledge and attitude among healthy individuals during the COVID-19 pandemic, we infer the following and report the results as per Behzadi and Gajdács [30].

**Figure 4:**
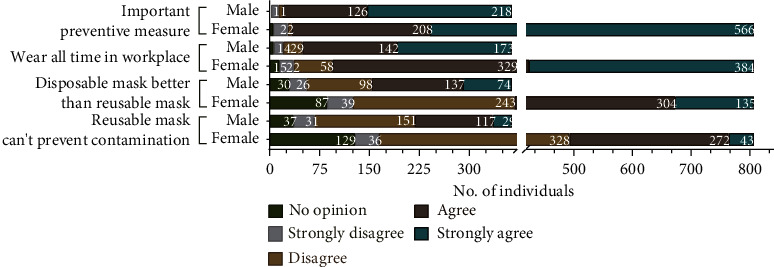
Awareness of mask wearing during the pandemic. The graph compares participants who agree, disagree, or have no opinion. Numbers inside the bar graphs represent total number of individuals against each opinion. Individual counts lower than eleven are not marked but is included in the bar graphs. Awareness or knowledge was tested for mask use as preventive measure; wearing mask at all time in workplace; comparing preference of disposable and reusable mask; and believing that reusable mask cannot prevent against contamination.

The survey consisted of participants majorly belonging to the younger population, that is, 20–30 years of age, while only 146 people (6.14% women and 6.31% men) were above 40 years of age (Table 2). 68.8% of the total participants (808 individuals) were female participants. With the majorly young population participating in this survey, this study could not relate them to any job specifications, barring us from correlating the mask wearing to any job demanding any physical effort. We assume that this young population is an active and healthy group (Figure 3) who have been still affected by this new way of life. 45.87% (368 women and 170 men) were found to be using fabric or cloth masks, followed by the use of surgical mask by 29.83% (251 women and 99 men) and N95 mask by 23.7% (183 women and 95 men) (Table 3). As a common perception, they felt disposable masks to be better than reusable masks and they are effective in preventing contamination during the pandemic (Figure 4). The disposal of such masks remains a big concern and a major scope of further discussion to be explored. Masks are strongly accepted by the general population as an important preventive measure during this pandemic and are preferred to be worn at work or outdoors. This indicates that the necessity, purpose, and knowledge of using mask have reached among the mass.

**Figure 5:**
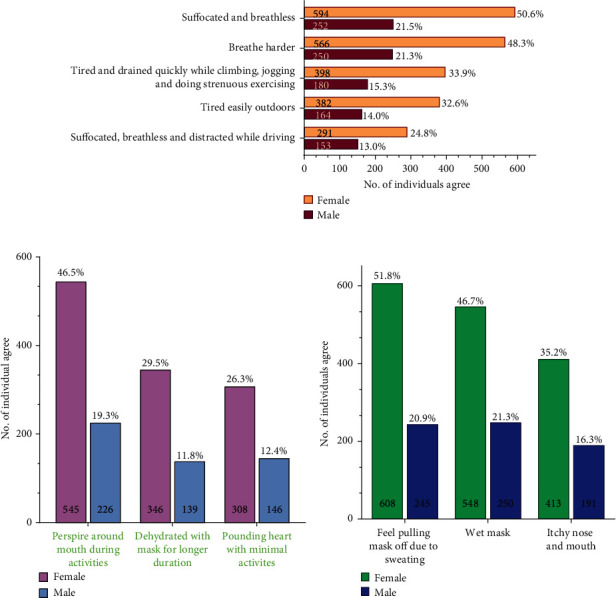
Impact and comfort of wearing mask. (a) This graph compares various impacts that wearing a mask can have. This includes feeling suffocated, breathless, and tired leading to need to breathe harder while outdoor, during exercise or driving. (b) This graph shows the comparison of impact and comfort wearing mask that includes perspiration around mouth, feeling dehydrated, and pounding heart. (c) Comparing percentage of participants who have wet mask, feel like pulling mask off, and feel itchy around mouth due to sweating while wearing mask. In all graphs, numbers inside the bar graphs represent total number of individuals that agree while numbers outside the bar graphs represent percentage of total participants.

A gender-based assessment of major perceptions of mask usage in public/work places is enlisted in Table 4. It was interesting to find that while a majority of 594 women and 252 men (72.1% participants) experienced suffocation in general, while using mask, only 291 women and 153 men (37.9% participants) felt suffocated while driving (Figure 5(a)). Comparatively, 382 women and 164 men (46.5% participants) feel tired outdoors in general similarly with 398 women and 180 men (49.3% participants) who feel tired with strenuous activities like jogging and climbing stairs (Figure 5(a)). However, we cannot conclude that women were overwhelmed with mask wearing as more women participated in this survey. On a large scale, 90.79% that includes 769 women and 296 men have refrained from disclosing their personal habits of smoking or alcohol consumption (Table 4), and hence, such habits cannot be correlated in this study as having any effects on the impact of wearing mask.

**Figure 6:**
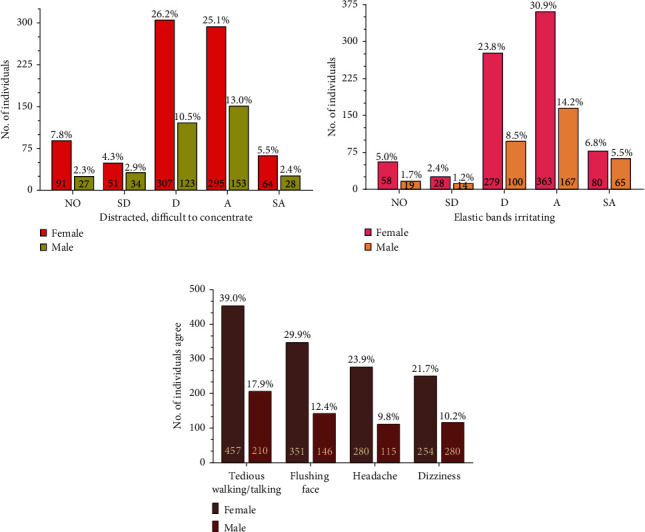
Mood and effects of wearing mask. (a) The graph compares percentage of participants who strongly agree (SA), agree (A), disagree (D), strongly disagree (SD), or have no opinion (NO) on feeling distracted and finding it difficult to focus while wearing mask. (b) The graph compares percentage of participants who strongly agree (SA), agree (A), disagree (D), strongly disagree (SD), or have no opinion (NO) that elastic bands on mask are irritating. (c) The graph compares effects of wearing mask for longer duration like headache, dizziness, flushing face, and tedious walking/talking. In all graphs, numbers inside the bar graphs represent total number of individuals that agree while numbers outside the bar graphs represent percentage of total participants.

In the survey questionnaire, along with the advantages and requirement of using mask frequently during the current pandemic situation, the participants pointed out a few drawbacks with regard to wearing mask and the hurdles they faced daily. Despite the disparity in the preference of mask, approximately two-thirds of the participants 541 women and 211 men found wearing them as uncomfortable (Table 4). It was interesting to observe that while 95.31% of the participants (774 women and 344 men) agreed that mask wearing is an effective preventative measure and 87.64% (713 women and 315 men) believed that should be religiously worn in workplaces, 69.6% (566 women and 250 men) of them were overwhelmed by the impact of wearing it with the feeling to breathe harder with mask worn (Figures 4 and 5(a)). Majority of the population voted that wearing mask makes them feel suffocated and breathless, due to which they feel the need to inhale air more than normal through their mouths and nostrils (Figure 5(a)). Wearing mask for longer duration and performing activities was known to cause increased perspiration, tiredness, and sweating around the nose and the mouth, which increases the urge to pull the mask off the face for gasps of air (Figures.  5(b), 5(c) and Table 4). 51.5% participants (413 women and 191 men) also felt that mask causes itchiness around the nose and the mouth irrespective of the type of mask used (Figure 5(c)). The elastic bands holding the mask to the face are uncomfortable and cause irritation and distraction (Figure 6(a), 6(b) and Table 4). It needs to be realized that not all masks will necessarily have elastic bands. Hence, 35.9% (307 women and 114 men) have disagreed on this aspect (Figure 6(b)). Further, people working as a health worker might use two different types of masks, at workplace and outdoors, but might have opted for only one option while taking our survey and reflected their knowledge and attitude accordingly.

38.7% (308 women and 146 men) participants believed that the heart beat increases even with minimal activities. This indicates that probably people do not pay attention to this matter and healthy persons are really not affected much (Figure 5(b)).

Only 42.3% of the participants (351 women and 146 men) had flushing effects on their face by long duration mask wear, while 15.35% of the total participants (130 women and 50 men) were not opinionated at all (Figure 6(c) and Table 4). Some of the male participants reached back mentioning that they cannot feel the flushing on face and had no idea about how that can be judged. This indicated a gender bias where probably women felt this phenomenon more naturally than men. Further studies or surveys can be performed to introspect this scenario. However, less than half of the participants reported having the flushing effects clearly indicating that this condition might not be a serious implication during the mask wearing.

**Figure 7:**
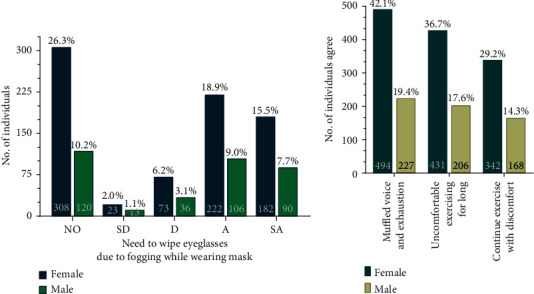
Effect on vision, voice, and exercising while wearing mask. (a) This graph compares percentage of participants that wear eye- or sun glasses and are affected by its fogging due to mask wearing while away from home. NO–no opinion, SD–strongly disagree, D–disagree, A–agree, ad SA–strongly agree. (b) This graph compares percentage of participants that agree on being conscious of their muffled voice and exhaustion, uncomfortable with long duration exercises and yet prefer to continue exercising to keep them healthy. In both graphs, numbers inside the bar graphs represent total number of individuals that agree while numbers outside the bar graphs represent percentage of total participants.

Due to increased perspiration, the mask causes the moist air from the nose to travel upwards towards the bridge of the nose and the eyes causing fogging of the glasses for people (51.1% participants including 404 women and 196 men) who wear spectacles or sunglasses on a regular basis (Figure 7(a) and Table 4).

**Figure 8:**
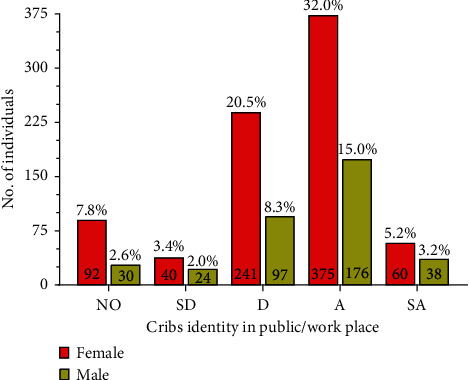
Mask wearing cribs identity in public/work place. The graph compares percentage of participants who strongly agree, agree, disagree, strongly disagree, or have no opinion on identity crisis. Numbers inside the bar graphs represent total number of individuals that agree while numbers outside the bar graphs represent percentage of total participants. NO–no opinion, SD–strongly disagree, D–disagree, A–agree, and SA–strongly agree.

Performing minimal physical activities, walking, climbing stairs, jogging, and shopping with mask on is difficult as compared to the normal days (Figure 5(a) and Table 4). They find it difficult to manage the mask when they are outdoors. 54% believed that identity crisis might become an issue and can lead to serious identity thefts since people are not keen on jeopardizing their health and safety by removing their masks in public to prove their identity (Figure 8 and Table 4). Interestingly, 435 women as compared to 214 men (57.63% participants) felt this identity crisis. 61.5% (494 women and 227 men) opinionated that their muffled voice require them to shout and exhaust them while wearing the mask, while 54.3% (431 women and 206 men) are uncomfortable exercising for longer duration (Figure 7(b) and Table 4). People exercising regularly have voted that they find it difficult to exercise for longer duration because the masks cause irritation, get wet due to the sweat, and cause breathlessness (Figures 5(c) and 7(b)). In spite of the practical difficulties while exercising with the mask on, they voted that they feel the need to exercise regardless of the uncomfortable feeling (Figure 7(b) and Table 4). This indicates a strong awareness among the general population regarding the need to exercise regularly. Exercising calms the mind and helps with warding off negative feeling and keeps us energized throughout the day.

Mann–Whitney test was performed for all graphs (Figure 5 to Figure 8), and no significant difference was observed between women and men on a *p*-value <0.05 except for Figures 5(a) and 6(c) that showed *p*-values of 0.0079 and 0.0286, respectively.

## 4. Discussion

Women and men responded very similarly when each gender's total percentage was calculated based on the total count of 808 women and 365 men, respectively, that can be seen in Table 5. Equal percentage of women and men agreed that they felt mask wearing made them tired while climbing, jogging, and doing strenuous exercises; it was tedious while walking or talking; mask got wet; they felt itchy around nose and mouth, and they had a muffled voice (Table 5). The overall health aspects of wearing mask and ways of improved lifestyles are discussed here.

Acute cardiovascular responses referred to as “responses” to exercise are the changes that occur in the body during and shortly after an exercise bout. Oxygen demand in the active muscles during exercise increases sharply, and more nutriments are needed. Metabolic processes accelerate generating more waste. During intense exercise, H+ concentration increases in the muscles and blood, lowering their pH [25, 26, 30]. This increase in the dependence on anaerobic metabolism corresponds to increase in blood lactate with increasing exercise intensity without the use of mask use. But the mask would cause the blood pH to decrease further, causing acidotic atmosphere in the body [31]. Cardiovascular changes during exercise allow the system to meet the higher demands placed on it and carry out its functions with maximal efficiency. There is an increase in heart rate with increase in intensity at a faster rate when exercising with mask than exercising without mask [32]. There is an increase in the peripheral vascular resistance, which increases the stroke volume thus increasing the cardiac output. The oxygen demand is met when exercising without the mask, although exercising with the mask causes retention of carbon dioxide in the mask, thus not synchronising with the normal mechanism leading to early fatigue and dyspnoea. Typically, 15%–20% resting cardiac output goes to muscles. During exhaustive exercise, the muscles receive 80%–85% of the cardiac output. Exercising with mask leads to increased metabolism causing increase in acidity, carbon dioxide, and temperature in the muscle tissues in abnormal proportions, altering the blood pressures as well [32–34]. During prolonged training or exercise in hot surroundings without masks, the person is at risk of dehydration, and more plasma volume is lost in sweating in an attempt to maintain body temperature, whereas when considered with mask, there is loss of sweat and perspiration even at normal levels. There is increasingly more loss of water from the body causing abnormal changes in the plasma volume [25, 30, 35]. Hemoconcentration increases erythrocyte concentration substantially by up to 20% to 25%. This increase elevates the carrying capacity of oxygen in the blood that is favourable during exercise and gives a distinct benefit at altitude at rest and during submaximal exercise [30–32].

There is evidence that high-intensity endurance exercise lasting more than 90 minutes can reduce the immunity, lasting 72 hours [32]. Individuals walking for 40 minutes per day at 70–75% of their VO2 max have reduced chances of acquiring common cold than those who do not exercise [33, 34].

During stress, 20–30 minutes of aerobic exercise is known to have calming effect and is known to last for several hours [36]. Yoga and Tai Chi being mind-body type of exercise that can help reduce stress as it involves concentration and improves breathing. Exercising at moderate intensities for 150–300 minutes/week with intervals in between the sessions is recommended to be beneficial during the COVID-19 pandemic. The cardiopulmonary physiological effect of masked exercise is lower compared with mask-free exercise. Hydration and transpiration are the most critical components for close monitoring [33, 37, 38].

It is a well-known fact that masks worn for longer hours at a stretch can cause hypoxemia and can affect our health [39]. A study on surgeons has shown that even an hour of wearing surgical masks elevate the pulse rates and lower the arterial pulsation oxygen saturation (SpO_2_), indicating a possibility of a large decrease of PaO_2_ (partial oxygen pressure of arterial blood) [38]. This effect is exacerbated at ages above 35 years in both men and women. Another study on end-stage renal disease patients showed that wearing N95 mask during haemodialysis to prevent SARS infection reduced PaO_2_ and resulted in hypoxemia in majority of the patients [39]. PaO_2_ < 50 mm Hg leads to hypoxemia. The oxyhaemoglobin (Oxy-Hb) dissociation curve follows a nonlinear sigmoid curve that varies with changes in PaO_2_. It is affected by various factors that include pH, temperature, PaCO_2,_ and 2,3-bisphosphoglycerate [40, 41]. For <80% SpO_2_, the resultant PaO_2_ can reduce to 40 mm Hg or lower that can lead to excessive hypoxemia [42]. It is also critical here to understand that hypoxemia and hypoxia are not identical and are related to blood and tissue oxygenation, respectively. Hence, they may or may not occur simultaneously and do have different pathophysiological effects. Health hazards of habitual long time wearing of masks during COVID-19 crisis can lead to hypoxemia due to low blood oxygen content and then may further lead to hypoxia if oxygen supply to tissues is compromised. Both these conditions, however, result in short of breath and faster heartbeat and may be enhanced during exercise along with the use of mask.

While intermittent hypoxia today is being considered as a nontherapeutic alternative to cardiovascular treatments [43], prolonged hypoxia due to long hours of mask usage can become a health hazard in the long run with increased number of cardiac cases observed. Anaemia and clots can induce hypoxic condition in specific organs like muscles, brain, and liver due to reduction in red blood cells and unavailability of oxygen [44–46]. This can ultimately cause cell death or apoptosis at the tissue and organ levels. Insufficient oxygen supply can affect the cellular respiration and culminate in cerebral hypoxia, stroke, and neuronal death. Chronic hypoxia can create inability to communicate due to brain damage. However, early detection can revert the situation with immediate and sufficient oxygen supply.

When we wear a mask as a regular practice in the current scenario, we are breathing in more and more of the exhaled CO_2_ that can affect the pH status of our tissues in lung and blood and then various organs that the blood is carried to. Heavy breathing also cannot cope up with the increased oxygen demands in such a situation and will require to adapt to anaerobic respiration because of crossing the anaerobic threshold. CO_2_ levels in exhalation exceeding 4–5% can lead to disorientation and even death [47]. Even though the likelihood of such an incident occurring is quite unlikely and it can typically be avoided, it is nevertheless essential to raise awareness of it. Hypoxia causes closure of potassium-gated channels and depolarization of cells. This further leads to vasoconstriction due to the constitutive opening of calcium-gated channels causing high blood pressure. [48].

Proper and regular breathing and physical exercises are essential to adapt the body to changes in the oxygen availability and should become a practice of life during the COVID-19 pandemic crisis. Uniform work/rest cycles are required to be adjusted based on individual's amount of physical work in the workplace and the amount of personal workout duration if any. This will enable individuals to cope up with the risks that mask wearing can bring. At the same time, we need to explore ways to improve mask designing in a way that can allow purified air to enter into the mask covered nose and mouth region filtering away the bacterial and viral particles. Filtering facepiece respirators (FFRs), air-purifying respirators (APR), and blower powered air-purifying respirators (PAPR) have their own disadvantages that need to be overcome in terms of rashes/oedema development, visual hindrance by fogging/dust/precipitates, and other discomforts [47]. Usage of some of these respirators might not be even feasible or cost-effective on a daily basis by the common mass population. For example, using a PAPR will add extra carrying weight and can relate to many ergonomic health problems.

## 5. Conclusion

We can avoid the health risks that wearing a mask during the COVID-19 crisis can bring about by engaging in deep breathing exercises as well as general mobility exercises. Our survey findings show that education and awareness regarding the importance and necessity to using masks as a measure of prevention and containment of the virus during the pandemic is effectively delivered through media, magazines, and newspapers, which has reached the general population. As we learn to cope with this crises, we may also develop better coping mechanisms including better masks and improvise lifestyle adjustments that will strengthen our immunity and establish an internal capacity for self-healing, making us more resilient.

## Figures and Tables

**Table 1:** List of some commonly available masks.

Sr. No.	Types of masks
1	Respirator masks [24–31]
2	N95 masks [32–40]
3	Surgical masks [37, 38, 40, 41]
4	Air purifier masks [42, 43]
5	Fabric face masks [32, 44–46]
6	Single-layer face masks [47]
7	Dust masks [47]

Note: Numbers in square brackets are reference numbers

**Table 2:** Age group-wise distribution of survey participants.

Age group	Women	Men
10–20	115	36
20–30	541	191
30–40	80	64
40–50	46	43
50–60	26	31
Total	808	365

**Table 3:** Preferred mask usage in public/work places.

I use this type of mask	Women	Men
Air purifier mask	1	0
Fabric/cloth mask	368	170
N95	183	95
No opinion	5	1
Surgical mask	251	99

**Table 4:** Gender-based experiences of mask use in public/work places.

Experiences of mask use	Women	Men
Use of cloth/fabric mask	368	170
Didn't disclose personal habits of smoking/alcohol	769	296
Uncomfortable wearing mask	541	211
Feel like pulling mask off	608	245
Flushing face	351	146
Itchy nose and mouth	413	191
Perspire around mouth	545	226
Dehydrated	346	139
Distraction	359	181
Elastic bands irritating	443	232
Dizziness	254	120
Pounding heart	308	146
Fogging eyeglasses	404	196
Identity thefts	426	208
Muffled voice and exhaustion	494	227
Exercise with uncomfortability	342	168
Difficult to exercise for long	431	206

**Table 5:** No gender biasness on feelings.

Feeling	Total women% agree	Total men% agree
Tired quickly while climbing, jogging and doing strenuous activities	49.3	49.3
Wet mask	67.8	68.5
Itchy nose and mouth	51.1	52.3
Tedious walking/talking	56.6	57.5
Muffled voice, exhaustion	61.1	62.2

## Data Availability

All relevant articles, book(s), and book chapter(s) have been included in the reference list. Raw data and material for the questionnaire are available on demand via proper channel to share maintaining participant anonymity.

## Additional Points

This article is written as per STROBE guidelines following STROBE checklist for cross-sectional studies.
